# Patient Selection and Reoperation After Cement-Augmented Pedicle Screw Fixation in the Thoracolumbar Spine: A Single-Center Cohort

**DOI:** 10.3390/jcm15207867

**Published:** 2026-10-12

**Authors:** Hershil A. Patel, Rohan I. Suresh, Anthony K. Chiu, Sapan Patel, Shivam Khanna, Joseph Mixa, Hans Prakash, Amil Sahai, Akul Patel, Alexander Padovano, Louis J. Bivona, Daniel L. Cavanaugh, Eugene Y. Koh, Steven C. Ludwig, Julio J. Jauregui

**Affiliations:** Division of Spine Surgery, Department of Orthopaedics, University of Maryland School of Medicine, Baltimore, MD 21021, USAskhanna@som.umaryland.edu (S.K.);

**Keywords:** thoracolumbar, cement augmentation, pedicle screws, bone quality, Hounsfield unit, opportunistic computed tomography, reoperation

## Abstract

**Background/Objectives:** Poor bone quality compromises pedicle screw fixation, and cement augmentation of fenestrated screws improves fixation at the screw–bone interface. Whether low interface-failure rates translate into low all-cause reoperation rates in routine practice is not established. We characterized the institutional use of cement-augmented fenestrated pedicle screws, the retrospectively measured vertebral computed tomography (CT) attenuation of selected patients, and subsequent interface failure and reoperation. **Methods:** We reviewed all patients undergoing thoracolumbar pedicle screw fixation between January 2016 and June 2022 at one tertiary referral center. Vertebral attenuation was measured retrospectively in Hounsfield units (HU) at L1 through L4. Outcome proportions are reported with Wilson 95% confidence intervals. Comparisons across surgical indications used exact tests with Holm correction and were treated as exploratory. Cement leakage and other cement-specific safety events could not be ascertained and were not study outcomes. **Results:** Cement augmentation was used in 101 of 2285 screened patients (4.4%), and 68 met the criteria for the outcome cohort (median follow-up, 44.9 weeks). Median attenuation was 94 HU, and 35 of 53 measurable patients (66.0%) fell below 110 HU, whereas formal densitometry was documented in 3. Screw loosening and pullout each occurred in three patients (4.4%), and reoperation for any cause occurred in nine (13.2%). Reoperation was more frequent among deformity constructs (30.4%) than for trauma (6.1%) or oncologic disease (0%); omnibus exact *p* = 0.016, although this result did not remain significant after Holm correction (adjusted *p* = 0.10). **Conclusions:** Cement augmentation was applied selectively, and retrospective CT measurements identified low vertebral attenuation in most measurable patients, despite infrequent formal densitometry. Failure at the screw–bone interface was infrequent, whereas all-cause reoperation remained clinically relevant and varied by surgical context. This study describes the selection and outcomes after augmentation but cannot establish comparative effectiveness or the safety of the augmentation technique.

## 1. Introduction

Poor bone quality compromises the fixation of pedicle screws. Reduced bone mineral density (BMD) is an independent predictor of screw loosening [1], is associated with lower rates of successful arthrodesis [2], and increases the risk of complications, including incidental durotomy [3]. When fixation fails at the screw–bone interface, the consequences extend beyond the screw itself and include loss of correction, pseudarthrosis, and reoperation. Osteoporosis is also underdiagnosed in patients undergoing lumbar fusion [4], so the patients at greatest risk are often not identified as such before surgery. Bone quality has therefore become a routine consideration in preoperative planning for an aging surgical population.

Cement augmentation of fenestrated screws is one response to this problem. Biomechanical studies show that augmentation increases the fatigue load and rigidity of the screw–bone interface in vertebrae with reduced density [5,6,7,8,9], and clinical series report low rates of loosening and hardware failure in augmented constructs [10,11,12,13]. These data address the interface directly. They do not describe how often the technique is used within an unselected operative population, in which patients are selected in practice, or whether a low rate of interface failure corresponds to a low rate of reoperation for any cause. Reoperation after instrumented fusion is driven by pseudarthrosis, symptomatic hardware, and adjacent pathology, as well as by screw loosening, and a construct can remain fixed at the screw while still requiring revisiting the operating room.

Dual-energy X-ray absorptiometry (DXA) is the reference standard for diagnosing osteoporosis, but many patients presenting after trauma or with metastatic disease do not have DXA available before surgery. In these patients, vertebral attenuation, measured in Hounsfield units (HU) on computed tomography (CT) obtained for other indications, has been proposed as an opportunistic surrogate for BMD [14]. HU values correlate with DXA results and with the compressive strength of bone, and values in the range of 110 to 135 have been proposed as reference thresholds below which bone quality warrants attention [14,15,16]. What remains unclear is the bone-quality profile of patients actually selected for augmentation in practice and whether their subsequent course is characterized by interface failure or by other mechanisms causing reoperation. The purpose of this study was to characterize institutional use of cement-augmented fenestrated pedicle screws, to describe the retrospectively measured vertebral CT attenuation of selected patients, and to evaluate interface failure, reoperation, and maintenance of alignment across surgical indications. We hypothesized that screw–bone interface failure would be infrequent, although all-cause reoperation would remain influenced by indication and construct complexity. Fixation failure is multifactorial, and construct mechanics, spinal alignment, and muscle quality contribute alongside bone quality. This study approaches the problem from the perspective of bone quality and the screw–bone interface. Cement-specific safety events, including cement leakage, could not be captured reliably in this retrospective cohort and were outside its scope.

## 2. Methods

### 2.1. Study Design and Patient Selection

This retrospective cohort study was approved by the institutional review board (HP-00071677). All patients older than 18 years who underwent thoracolumbar pedicle screw fixation between 1 January 2016 and 1 June 2022 at one tertiary referral center were screened to identify those who received cement-augmented fenestrated pedicle screws. Patients were excluded if radiographic imaging was not available for at least 2 weeks postoperatively or if there was active infection at the operative site at the time of surgery. Of 101 augmented patients, 68 met the criteria and form the analytic cohort (Figure 1). Cement-augmented fenestrated pedicle screws were placed at the discretion of the treating surgeon, and polymethylmethacrylate was injected through the screw shaft under fluoroscopic visualization. Procedures were performed by multiple attending spine surgeons within one division. Because this was a review of routine practice, the implant system and cement product were not prescribed by protocol and were not recorded in a manner that permitted systematic capture.

Demographic, intraoperative, and postoperative data were abstracted from the medical record, including the number of levels instrumented, surgical approach, operative time, length of stay, estimated blood loss, discharge disposition, readmission, and reoperation. The term reoperation rather than revision is used throughout because the recorded indications were heterogeneous and included procedures that did not revise the index construct. Every outcome denominator reported here is for the analytic cohort of 68 patients, except vital status, which uses the 61 patients for whom vital status was recorded. Because inclusion required postoperative imaging, patients who died early or received subsequent care elsewhere may be underrepresented, and this is addressed in the Limitations. Baseline data were abstracted only for patients who met the inclusion criteria, so the 33 excluded patients could not be compared with the analytic cohort. Cement-specific safety outcomes, including cement leakage or extravasation, pulmonary cement embolism, and cement-related neurologic injury, were not study outcomes. Follow-up relied on plain radiographs and the clinical record, postoperative CT was not obtained systematically, and cement volume was not recorded consistently, so these events could not be reliably ascertained.

### 2.2. Radiographic Assessment

A spine surgery fellow reviewed all available radiographs for screw loosening and gross hardware pullout or failure. We defined screw loosening as the presence of a radiolucent rim greater than 1 mm around one or more screws [17]. Screw pullout was defined as gross migration of a screw from its intended position, and any screw-related problem was defined as a composite of loosening, pullout, or hardware failure occurring in the same patient. Patients who received short-segment instrumentation for thoracolumbar trauma or percutaneous stabilization for metastatic disease were additionally assessed for maintenance of alignment by measuring the regional Cobb angle across the instrumented levels, calculated within patients against the immediate postoperative radiograph, with negative values indicating loss of correction toward preoperative alignment. Measurements were performed by a single reviewer, and reliability testing was not performed.

### 2.3. Vertebral Attenuation Measurement

In patients for whom a preoperative lumbar CT was available within 1 year of surgery, we measured attenuation in HU at the L1 through L4 vertebral bodies using a previously described method [15] (Figure 2). We excluded measurements where a preoperative CT was unavailable or where prior hardware, cement, or fracture level precluded accurate measurement, and we used a per-patient mean across measurable levels for cohort-level summaries. These measurements were performed retrospectively for this study and were not available to the operating surgeon at the time of the decision to augment. Examinations were obtained clinically over a 6-year period, so contrast state and acquisition parameters were not standardized. Attenuation was recorded as displayed on the clinical examination, and we did not apply phantom calibration or a correction for tube voltage or contrast phase.

### 2.4. Statistical Analysis

Continuous variables are reported as mean ± standard deviation or median with interquartile range (IQR), and categorical variables as counts and percentages. Outcome proportions are presented with Wilson 95% confidence intervals (CI). Categorical variables were compared using the Fisher exact test, extended to the exact conditional test of Freeman and Halton for comparisons across the three indication groups. The Chi-square test was not used for these comparisons because at least one expected cell count was below 5 in each comparison, which invalidates the approximation. Because six outcomes were compared across indications, we applied Holm–Bonferroni correction to that family and report both raw and adjusted *p* values. Continuous variables were compared using the Mann–Whitney U test, and attenuation was compared across indications using the Kruskal–Wallis test.

Freedom from reoperation and freedom from radiographic screw loosening were estimated using the Kaplan–Meier method with log–log confidence intervals, censoring at the last available follow-up. Death was treated as censoring rather than as a competing event. Given the small number of observed mechanical events, we did not undertake multivariable modeling, and all comparisons across surgical indications are exploratory rather than confirmatory. A two-sided *p* value below 0.05 was considered significant, and analyses were performed in Python 3 (NumPy, pandas, and SciPy).

## 3. Results

### 3.1. Institutional Use and Cohort Characteristics

Between 2016 and 2022, 2285 patients underwent thoracolumbar pedicle screw fixation, and cement augmentation was used in 101 (4.4%, 95% CI 3.7 to 5.3). After exclusions, 68 patients formed the analytic cohort (Figure 1). Median follow-up was 44.9 weeks (IQR 27 to 68), 20 patients had at least 12 months of imaging, and 4 had at least 24 months.

The cohort was elderly and medically complex (Table 1), with a mean age of 70.2 ± 9.9 years, with 48 female patients (70.6%), a mean Charlson Comorbidity Index of 2.6 ± 2.2, and ASA class III in 56 (82.4%) patients. Surgical indications were trauma in 33 patients (48.5%), deformity in 23 (33.8%), oncologic or metastatic disease in 11 (16.2%), and infection in 1 (1.5%). Constructs were posterior-only in 65 patients (95.6%), with a mean of 5.1 ± 2.9 instrumented levels. Fifty-five patients (80.9%) were treated with open surgery, and 13 (19.1%) using a minimally invasive technique. All deformity constructs were treated with open surgery.

### 3.2. Vertebral CT Attenuation and Documented Bone-Quality Testing

Preoperative CT permitted attenuation measurement in 53 of 68 patients. Among these, the median per-patient mean value was 94 HU (IQR 80 to 118). Thirty-five of 53 patients (66.0%) fell below 110 HU, and 42% fell below 88 HU (Figure 2). Attenuation did not differ across surgical indications (Kruskal–Wallis *p* = 0.99, Supplementary Table S1). Formal DXA was documented in three patients, and osteoporosis was coded in four.

### 3.3. Screw–Bone Interface Failure

Three of 68 patients developed radiographic screw loosening (4.4%, 95% CI 1.5 to 12.2), and 3 of 68 developed screw pullout (4.4%, 95% CI 1.5 to 12.2). Neither differed across surgical indications (loosening exact *p* = 0.75, pullout exact *p* = 1.00, Table 2). With three events per outcome, these comparisons had minimal power, and the absence of a statistically significant difference does not establish equivalence across indications.

Median attenuation was 102 HU among the 5 patients with a mechanical complication and 94 HU among the 48 without (*p* = 0.78, Supplementary Table S1). This comparison rests on five events.

### 3.4. Reoperation and Event Timing

Nine of 68 patients required reoperation for any cause (13.2%, 95% CI 7.1 to 23.3). Recorded indications were symptomatic hardware in nine, hardware failure in three, pseudarthrosis in three, neurologic compromise in two, and infection in one, with some patients contributing more than one indication (Table 3). Four patients were readmitted within 30 days (5.9%), and all-cause mortality within the study window was 8 of 61 patients with recorded vital status (13.1%).

According to Kaplan–Meier estimation, freedom from reoperation was 87% (95% CI 75 to 94) at 12 months, and freedom from radiographic screw loosening was 97% (95% CI 88 to 99) at 12 months (Figure 3). Six of the nine reoperations occurred within the first 6 months. Fewer than 10 patients remained at risk after 14 months, and estimates beyond that interval are not reported.

### 3.5. Outcomes by Surgical Indication

Seven of 23 deformity patients underwent reoperation for any cause (30.4%, 95% CI 15.6 to 50.9), compared with 2 of 33 trauma patients (6.1%) and 0 of 11 oncologic patients (omnibus exact *p* = 0.016, Table 2, Figure 4). After Holm correction across the six comparisons in Table 2, this omnibus comparison was not significant (adjusted *p* = 0.10). A pairwise comparison of deformity against all other indications combined gave 30.4% versus 4.4%, a risk difference of 26.0 percentage points (95% CI 7.9 to 46.7, exact *p* = 0.005). This pairwise contrast was performed post hoc and was not included in the Holm correction family, so the adjusted value above applies to the omnibus test and not to this comparison. All comparisons across indications are exploratory.

Deformity constructs were treated exclusively through an open approach, so surgical indication and operative approach are not separable in this cohort.

### 3.6. Regional Alignment in the Trauma Subgroup

Among trauma patients with imaging at each interval, the mean within-patient change in regional Cobb angle from the immediate postoperative baseline was −2.1° at 2 weeks (n = 19), +1.7° at 6 weeks (n = 20), −4.4° at 12 weeks (n = 15), −6.8° at 6 months (n = 18), and −9.0° at 12 months (n = 14) (Figure 5). Negative values indicate loss of correction toward preoperative alignment. The confidence interval excluded zero at 12 weeks, 6 months, and 12 months. Mean loss of regional alignment was observed from 12 weeks through 12 months. Different patients contributed measurements at each interval, so these values describe the patients imaged at each timepoint and are not serial measurements within a fixed group.

### 3.7. Descriptive Summary by Surgical Approach (Exploratory)

Estimated blood loss was lower in the minimally invasive group (median 90 versus 400 mL, *p* < 0.001), operative time was shorter (median 109 versus 217 min, *p* < 0.001), and length of stay did not differ (*p* = 0.57) (Table 4, Supplementary Figure S1). These values are descriptive and exploratory. The groups were not assembled by design and represent different patient populations and procedures, so the *p* values do not support a comparative inference between approaches. Because the approach followed the surgical indications, these differ in diagnosis, construct length, and operative goal, as well as in exposure. Rates of loosening, pullout, reoperation, and readmission did not differ significantly between approaches, with small event numbers in both groups.

## 4. Discussion

Cement augmentation was used in 4.4% of thoracolumbar pedicle screw constructs at this center, and the patients selected for it had low vertebral attenuation when measured retrospectively. Retrospective HU measurements identified low vertebral attenuation in most measurable patients, despite sparse formal densitometry, with DXA documented in three patients, and osteoporosis coded in four. This pattern is consistent with reports that osteoporosis is underdiagnosed in spine surgical candidates [4] and supports the use of opportunistic CT to characterize bone quality when DXA is unavailable [14,15]. Two limits on this inference deserve emphasis. HU values were measured for this study and were not available at the time of the operative decision, so they describe the patients selected but do not describe the basis on which they were chosen. Similar HU distributions across indication subgroups suggest that poor bone quality was not confined to a single diagnostic category, though without measurements in nonaugmented patients, the study cannot describe how augmented and nonaugmented patients differed.

Screw loosening and pullout each occurred in 4.4% of patients, whereas all-cause reoperation occurred in 13.2%, and most events occurred within the first 6 months. The interface-failure rates sit within the published range for augmented constructs. Kim et al. reported that cement augmentation reduced loosening from 50% to 7.3%, with fenestrated screws outperforming solid screws [10], and Ehresman et al. reported a 2.6% rate of asymptomatic loosening [11]. Ghermandi et al. described comparable durability in a series of patients with low bone quality treated with augmented fenestrated screws [12]. In metastatic disease, Wagner et al. reported an 11.8% loosening, with no pullout [13], and a recent meta-analysis estimated a pooled hardware-failure rate of 4.3% [18]. What the present cohort adds is the reoperation rate observed alongside those low interface-failure rates, and the recorded indications were dominated by symptomatic hardware and pseudarthrosis, not by loosening. Archie et al. catalogued the modalities by which augmented screws fail and drew attention to failure occurring away from the interface [19]; the present findings are consistent with that emphasis. A construct may remain fixed at the screw while the patient still requires a return to the operating room, and series reporting only loosening will understate the reoperation burden.

Reoperation was concentrated among deformity constructs, occurring in 30.4% of deformity patients compared with 6.1% of trauma patients and none of the oncologic patients, although this difference did not remain significant after correction for multiple comparisons and should be read as hypothesis-generating. Deformity constructs in this cohort were longer, crossed more mobile segments, and carried a higher baseline reoperation risk irrespective of augmentation, so confounding by indication is the most plausible explanation. Mean loss of regional alignment from 12 weeks through 12 months in the trauma subgroup points in a similar direction. Prior reports describe augmented constructs maintaining reduction in osteoporotic and traumatic fractures [20,21,22,23,24], and a construct can hold at the screw while the segment loses correction through the disc, the endplate, or an adjacent level. The decreased blood loss and shorter operative time observed with minimally invasive placement are consistent with the broader minimally invasive literature for thoracolumbar trauma [25,26], but this summary is descriptive and ancillary. Since approach followed indication, and since the deformity patients who were treated with open surgery also accounted for most reoperations, the operative advantage of a minimally invasive corridor and the reoperation burden of deformity surgery are not independent observations here.

Bone quality is one determinant of fixation failure among several, and this study was deliberately framed around it. Screw loosening has also been associated with the number of levels fused [27,28] and, at the lumbosacral junction, with spinopelvic mismatch and degeneration of the paraspinal musculature [28]. Sagittal alignment after adult deformity correction predicts mechanical complications [29], and open pedicle screw fixation is followed by greater multifidus atrophy than that for percutaneous fixation [30], so open exposure may reduce the muscular support of the instrumented segments. We did not measure sagittal alignment, rod contour, sarcopenia, or paraspinal muscle quality, and we did not analyze constructs by the junctions they crossed. Construct length and open exposure were also collinear with a deformity indication in this cohort. The findings should therefore be read from the perspective of bone quality and the screw–bone interface, and the higher reoperation rate among long, open deformity constructs is consistent with a contribution from these mechanical and muscular factors that the present data cannot separate from indication.

Several limitations qualify these findings. The study is retrospective and drawn from a single center. Thirty-three of 101 augmented patients were excluded for insufficient postoperative imaging, and because this criterion is imaging-based, it may have removed patients who died early, were lost to follow-up, or returned elsewhere, which raises the possibility of attrition and selection bias in the reoperation denominator. Baseline characteristics were not abstracted for the excluded patients, so we could not compare the included and excluded groups, and the direction of any resulting bias is unknown. The cohort spans heterogeneous indications and construct lengths, and indication, construct complexity, and approach are collinear, so confounding by indication cannot be separated from the effect of any one of these. The number of mechanical events is too small for multivariable adjustment, so no adjusted estimate of the deformity association is available, and comparisons across indication are exploratory. The study lacks an uncemented comparison group, so it describes selection and outcomes after augmentation but does not establish comparative effectiveness. Radiographic follow-up was variable and relatively short, fewer than 10 patients remained at risk beyond 14 months, and death was treated as censoring instead of as a competing event in a cohort with 13.1% in-window mortality. Ascertainment of pseudarthrosis was not standardized. Different patients contributed Cobb measurements at each interval, so those values describe the patients imaged at each interval and not a fixed group followed serially. Reliability testing was not performed for HU, loosening, or Cobb measurements, and because examinations were obtained clinically across a 6-year period, CT contrast state and acquisition parameters, neither of which can shift attenuation values, were not standardized. Attenuation values were not phantom-calibrated or corrected for these differences. Regional Cobb angles were measured on plain radiographs by a single reviewer, so measurement error from patient positioning, radiographic projection, and endplate identification cannot be quantified, and the smaller changes observed at early intervals should be interpreted with caution. Procedures were performed by multiple attending surgeons, and the small number of events did not permit a surgeon-level analysis, so an effect of the individual surgeon on complication or reoperation rates cannot be excluded. Finally, implant system, cement volume per screw, the number of screws augmented, and the specific levels augmented were not recorded in a manner that permitted systematic capture, so we cannot confirm that all patients received implants and cement from a single manufacturer. Cement-specific complications, including leakage or extravasation, pulmonary cement embolism, and cement-related neurologic injury, were not assessed. This cohort, therefore, speaks to the outcomes of augmented constructs and not to the safety of the augmentation technique itself, which remains the more common concern in the existing literature.

## 5. Conclusions

At this center, cement augmentation was used selectively, and retrospective CT measurements identified low vertebral attenuation in most measurable patients, despite infrequent formal DXA documentation. Screw–bone interface failure was uncommon, whereas all-cause reoperation remained clinically relevant and varied by surgical context, with the observed deformity association remaining exploratory after multiplicity correction. These findings support further evaluation of standardized opportunistic CT assessment and controlled comparisons of augmented and unaugmented fixation stratified by surgical indication.

## Figures and Tables

**Figure 1:**
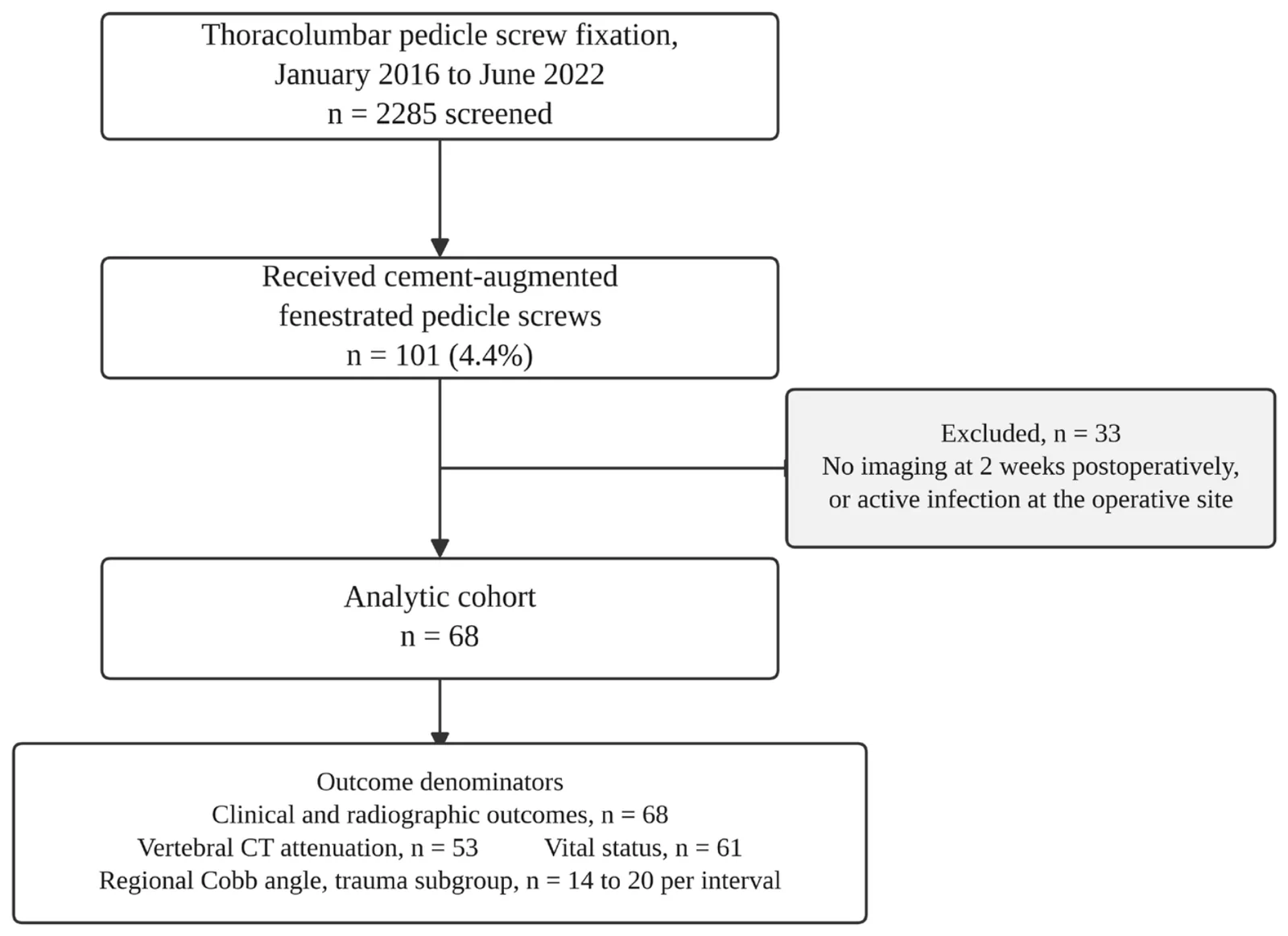
Patient selection. Of 2285 patients screened, 101 received cement-augmented fenestrated pedicle screws, and 68 met the criteria for the analytic cohort. Vertebral CT attenuation was measurable in 53 of these 68 patients.

**Figure 2:**
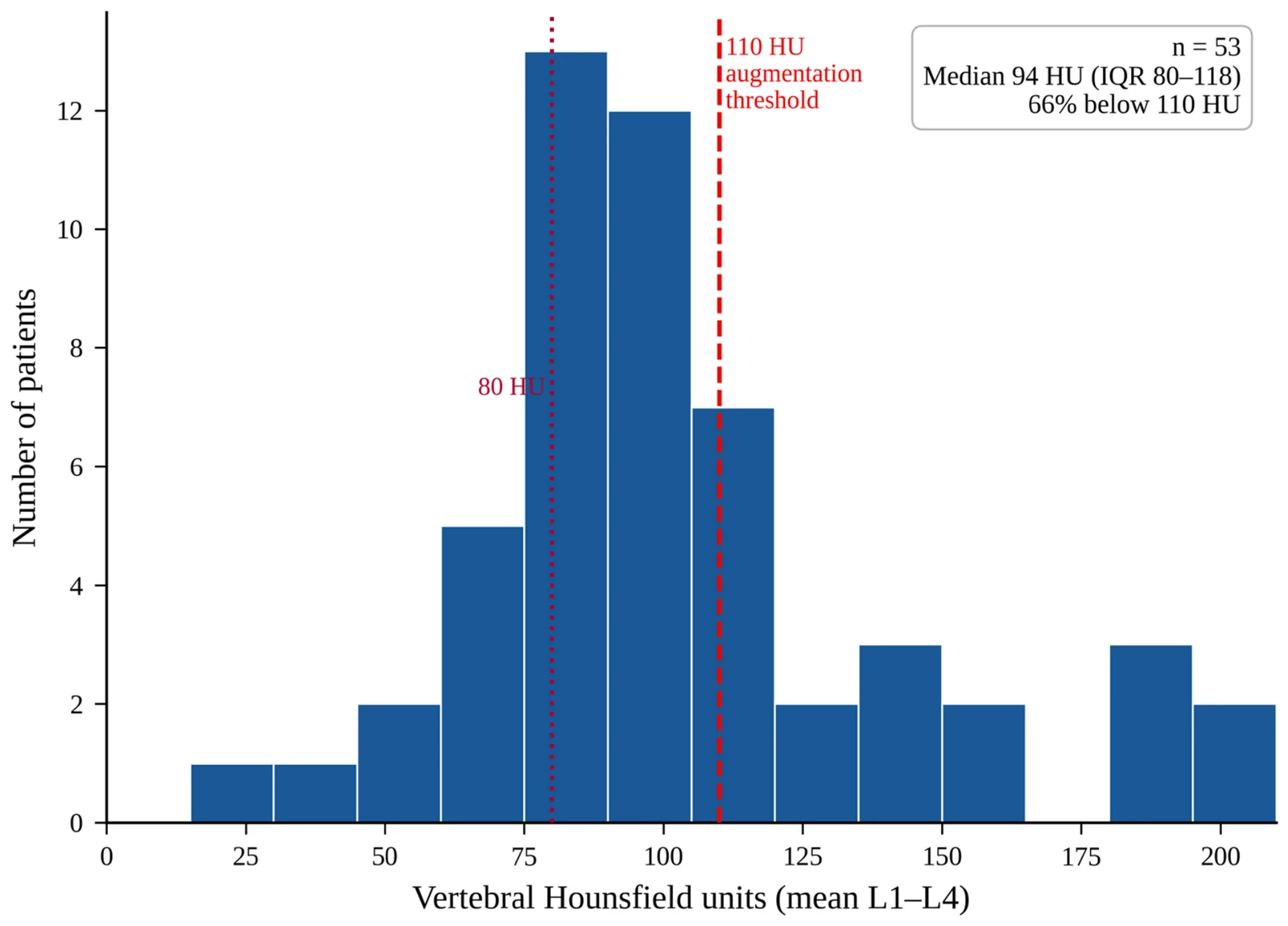
Distribution of preoperative vertebral attenuation (per-patient mean across L1 through L4) in 53 patients. The dashed line marks 110 HU, and the dotted line marks 80 HU, both reported as reference values for low vertebral attenuation.

**Figure 3:**
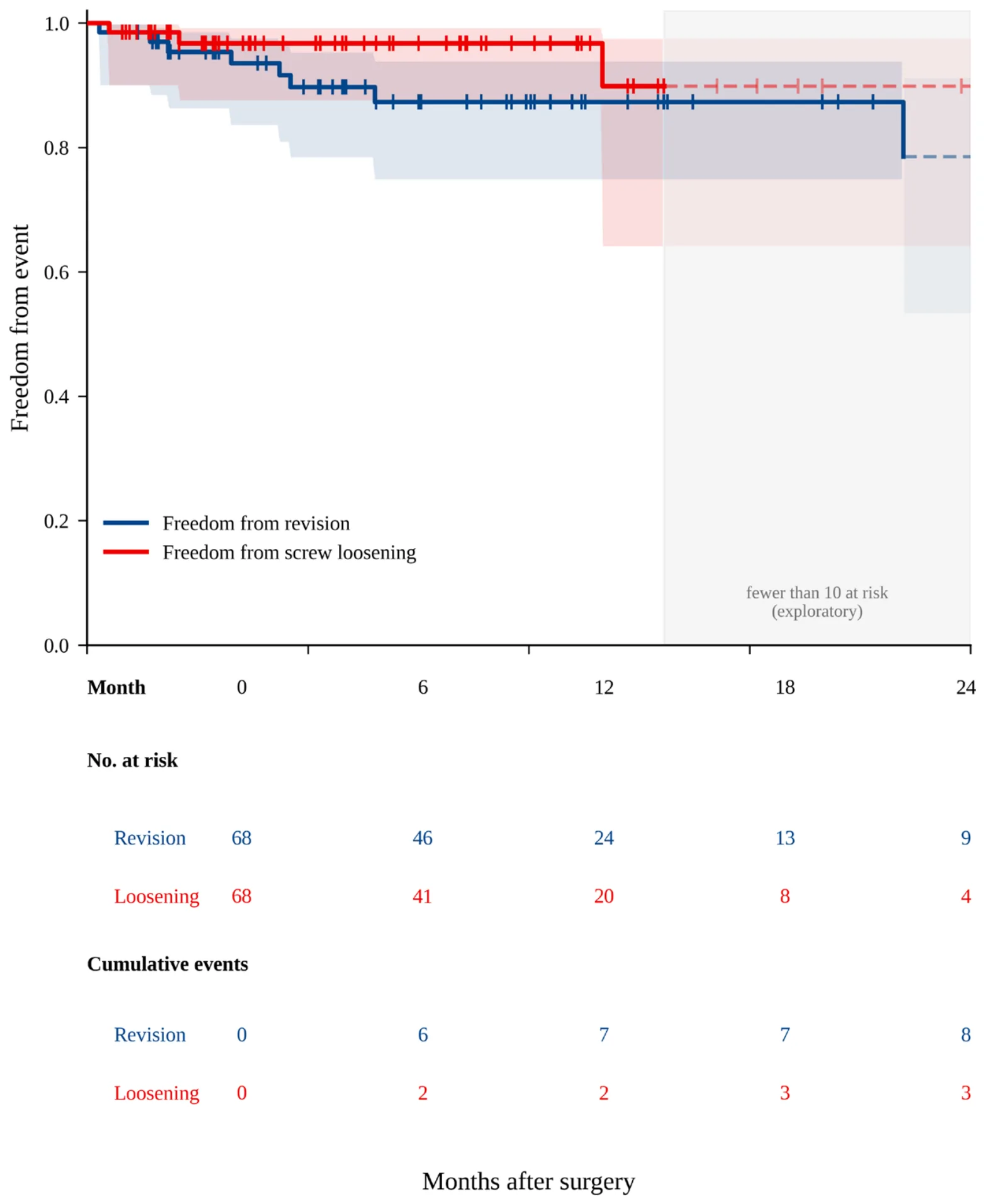
Kaplan–Meier curves for freedom from revision and screw loosening following surgery. Kaplan–Meier estimates of freedom from reoperation (blue) and freedom from radiographic screw loosening (red). Shaded bands are 95% confidence intervals, and vertical ticks denote censored patients. The number at risk and cumulative events are shown below the time axis. Solid lines indicate the portion of each curve during which at least 10 patients remained at risk for that outcome; dashed lines indicate estimates beyond that point, which are exploratory. The shaded region marks the interval in which fewer than 10 patients remained at risk for at least one outcome.

**Figure 4:**
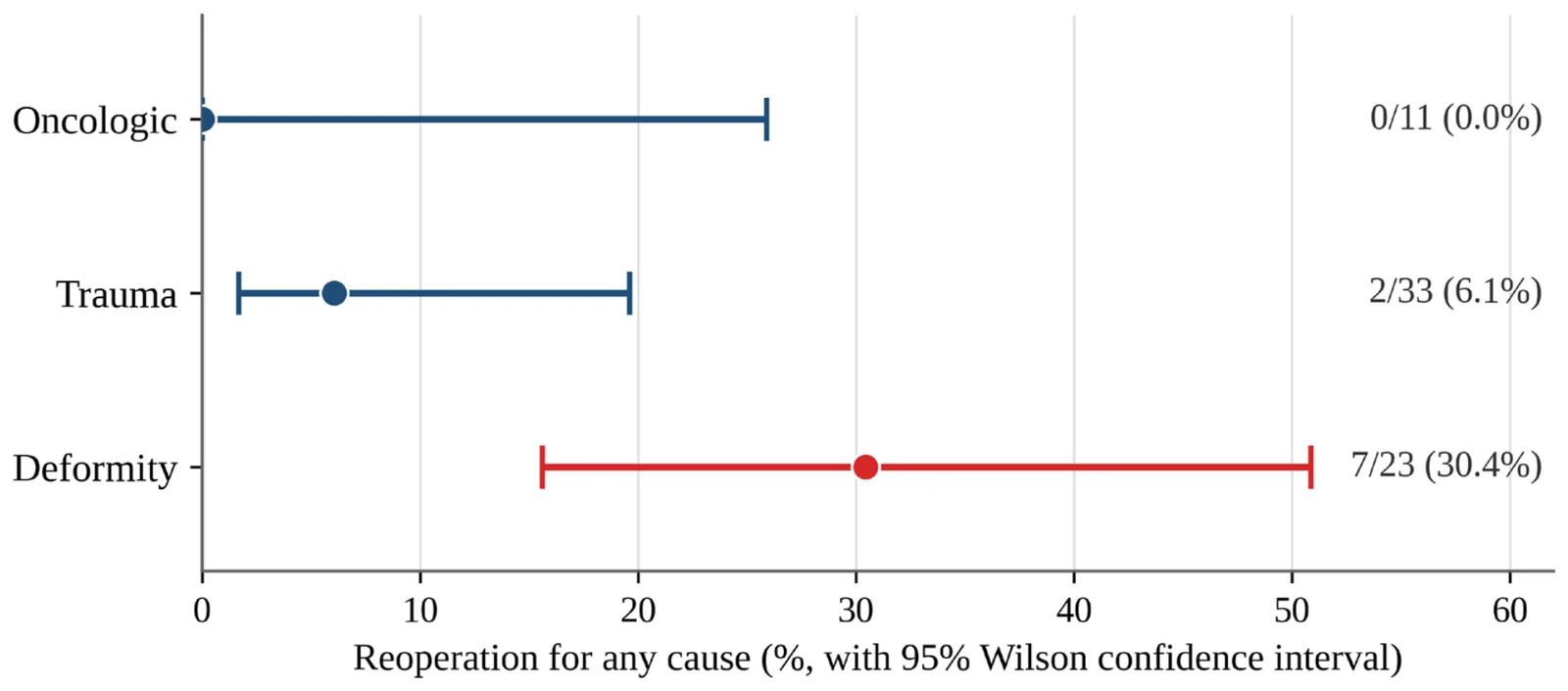
Reoperation rates by surgical indication. Reoperation for any cause by surgical indication, with Wilson 95% confidence intervals. The single patient treated for infection is not shown.

**Figure 5:**
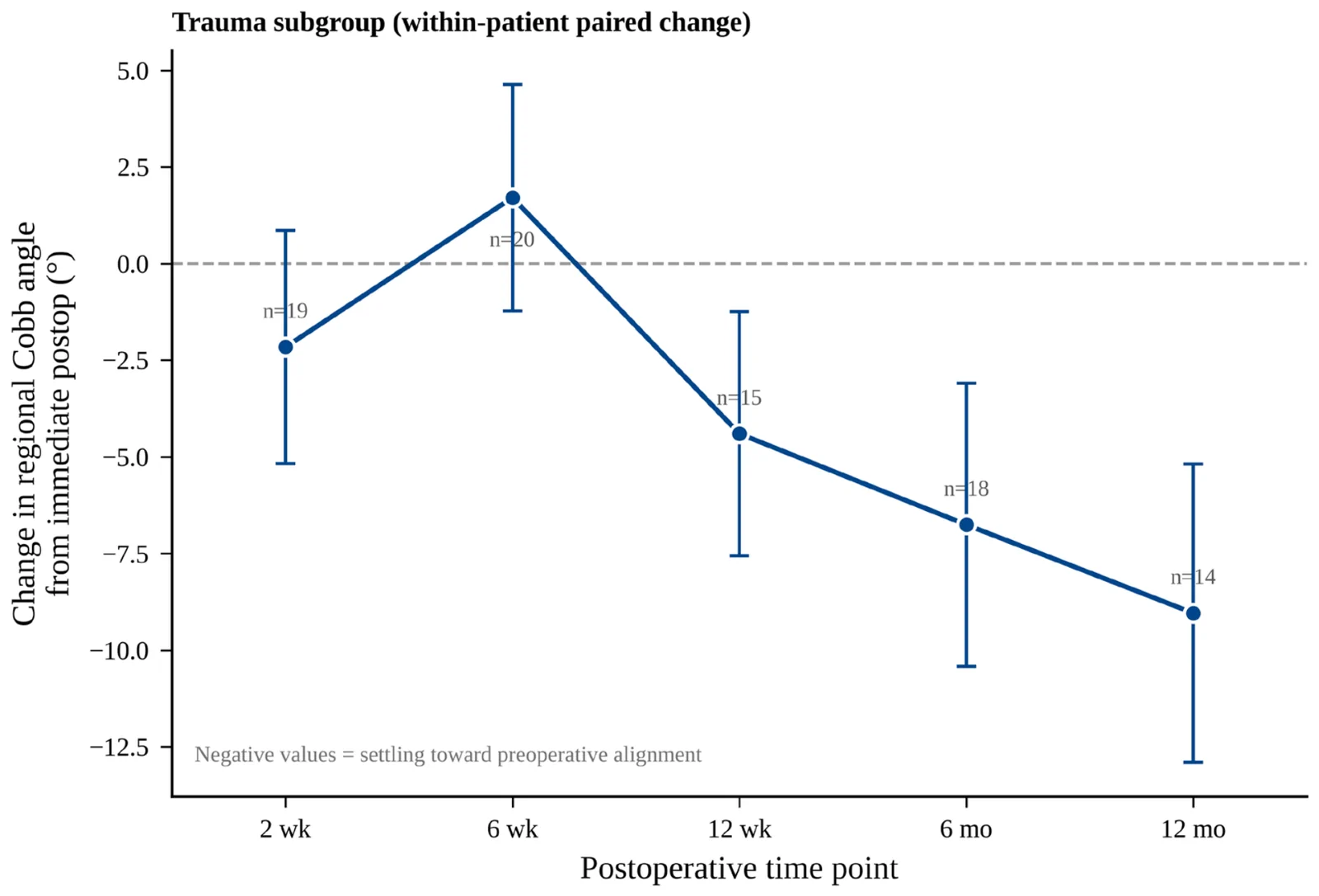
Longitudinal change in regional Cobb angle following surgery in the trauma subgroup. Mean within-patient change in regional Cobb angle from the immediate postoperative baseline among trauma patients with imaging at each interval. Points are the mean change with 95% confidence intervals, and the number of patients contributing at each interval is annotated. Negative values indicate loss of correction toward preoperative alignment. Different patients contributed at each interval.

**Table 1:** Baseline patient characteristics (n = 68).

Characteristic	Value
**Demographics**	
Age, yr, mean ± SD	70.2 ± 9.9
Female, n (%)	48 (70.6)
Body mass index, kg/m^2^, mean ± SD	29.6 ± 6.9
Current/former smoker, n (%) of 67	29 (43.3)/6 (9.0)
**Comorbidity**	
Charlson Comorbidity Index, mean ± SD	2.6 ± 2.2
ASA class III, n (%)	56 (82.4)
Diabetes mellitus, n (%)	18 (26.5)
Congestive heart failure, n (%)	13 (19.1)
Chronic kidney disease, n (%)	11 (16.2)
**Surgical indication**	
Trauma, n (%)	33 (48.5)
Deformity, n (%)	23 (33.8)
Oncologic or metastatic, n (%)	11 (16.2)
Infection, n (%)	1 (1.5)
**Bone quality**	
Mean L1-L4 attenuation, HU, median [IQR] (n = 53)	94 [80–118]
Attenuation < 110 HU, n (%) of 53	35 (66.0)
DXA documented/osteoporosis coded, n	3/4
**Follow-up and mortality**	
Follow-up, wk, median [IQR]	45 [27–68]
Died during follow-up, n (%) of 61	8 (13.1)

Values are mean ± standard deviation, median [interquartile range], or n (%). Percentages for smoking, attenuation, and mortality use available denominators (n = 67, 53, and 61, respectively), which are stated in the row labels. ASA, American Society of Anesthesiologists. DXA, dual-energy X-ray absorptiometry. HU, Hounsfield units. IQR, interquartile range.

**Table 2:** Outcomes by surgical indication (exploratory).

Outcome	Deformity (n = 23)	Oncologic (n = 11)	Trauma (n = 33)	Exact *p*	Holm *p*
Reoperation, any cause	7 (30.4)	0 (0.0)	2 (6.1)	0.016	0.10
Symptomatic hardware	4 (17.4)	0 (0.0)	1 (3.0)	0.107	0.53
Neurologic deficit	2 (8.7)	0 (0.0)	0 (0.0)	0.254	1.00
Screw loosening	2 (8.7)	0 (0.0)	1 (3.0)	0.746	1.00
Pseudarthrosis	2 (8.7)	0 (0.0)	1 (3.0)	0.746	1.00
Screw pullout	1 (4.3)	0 (0.0)	2 (6.1)	1.000	1.00

Values are n (%). Exact *p* from the Fisher exact test extended to the Freeman–Halton exact conditional test across three groups. The Chi-square test is not reported because at least one expected cell count was below 5 in each comparison. Holm *p* is the Holm–Bonferroni-adjusted value across the six omnibus comparisons in this table. No comparison remained significant after correction, and no raw *p* value is emphasized for that reason. The single patient treated for infection is not shown as a separate indication and had no reoperation. A pairwise comparison of deformity against all other indications for reoperation gave 30.4% versus 4.4%, a risk difference of 26.0 percentage points (95% CI 7.9 to 46.7), and an exact *p* = 0.005. This pairwise contrast was performed post hoc and was not part of the Holm correction family.

**Table 3:** Clinical and radiographic outcomes in the analytic cohort (n = 68).

Outcome	n (%)	95% CI
**Screw–bone interface**		
Screw loosening	3 (4.4)	1.5 to 12.2
Screw pullout	3 (4.4)	1.5 to 12.2
Any screw-related problem	8 (11.8)	6.1 to 21.5
**Reoperation and recorded indications**		
Reoperation, any cause	9 (13.2)	7.1 to 23.3
Symptomatic hardware	5 (7.4)	3.2 to 16.1
Hardware failure	3 (4.4)	1.5 to 12.2
Pseudarthrosis	3 (4.4)	1.5 to 12.2
Neurologic deficit	2 (2.9)	0.8 to 10.1
Surgical site infection	1 (1.5)	0.3 to 7.9
**Other**		
30-day readmission	4 (5.9)	2.3 to 14.2
Adjacent-segment disease/subsidence	0 (0.0)	0.0 to 5.3
Death, of 61 with recorded vital status	8 (13.1)	6.8 to 23.8

Wilson 95% confidence intervals. All denominators are obtained from the analytic cohort of 68 patients except for death, which uses the 61 patients with recorded vital status. Reoperation indications are not mutually exclusive and sum to more than nine. Any screw-related problem is the composite of loosening, pullout, or hardware failure, as defined in Methods 2.2. CI, confidence interval.

**Table 4:** Surgical and hospitalization characteristics, overall and by approach (descriptive and exploratory).

Characteristic	Overall (n = 68)	Open (n = 55)	MIS (n = 13)	*p*
Levels instrumented, median [IQR]	4 [4–6]	5	4	0.415
Operative time, min, median [IQR]	214 [157–260]	217	109	<0.001
n contributing [150–612]	64	53	11	
Estimated blood loss, mL, median [IQR]	350 [150–612]	400	90	<0.001
n contributing	68	55	13	
Length of stay, d, median [IQR]	6 [5–9]	6	8	0.567
Interbody cage, n (%)	6 (8.8)	-	-	-
**Discharge disposition, n (%)**				
Skilled nursing facility	35 (51.5)			
Home	30 (44.1)			
Acute rehabilitation	3 (4.4)			

Continuous variables compared by Mann–Whitney U test. *p* values are descriptive and exploratory and do not constitute a comparison of approaches. Approach was determined by surgical indication, and all deformity constructs were treated with open surgery, so these groups differ by diagnosis and construct, as well as by approach. Operative time was recorded for 53 open and 11 minimally invasive surgical patients, which is why those denominators are smaller than the column headers. Group medians are reported without interquartile ranges. IQR, interquartile range. MIS, minimally invasive surgery.

## Data Availability

Data are available upon request from the authors.

## Supplementary Materials

The following supporting information can be downloaded at: https://www.mdpi.com/article/10.3390/jcm15207867/s1, Supplementary Table S1. Group medians of patient-level mean L1–L4 attenuation by indication, approach, and complication status. Supplementary Figure S1. Operative metrics by surgical approach.

## Abbreviations

The following abbreviations are used in this manuscript:

ASA	American Society of Anesthesiologists
BMD	Bone Mineral Density
CT	Computed Tomography
DXA	Dual-Energy X-Ray Absorptiometry
HU	Hounsfield Units
MIS	Minimally Invasive Surgery
CI	Confidence Interval
IQR	Interquartile Range
SD	Standard Deviation
