# Supplementary material for: Patient Selection and Reoperation After Cement-Augmented Pedicle Screw Fixation in the Thoracolumbar Spine: A Single-Center Cohort
Source: J Clin Med. 2026 Oct 12;15(20):7867. doi: 10.3390/jcm15207867 (PMC13705460; doi:10.3390/jcm15207867)
Supplement: Supplementary file 1 [file jcm-15-07867-s001.zip › jcm-4557147-supplementary.pdf]

Supplementary Table S1. Group medians of patient-level mean L1–L4 attenuation, by indication, approach, and complication status.

Stratum	Median HU (n)	*p*
**By indication**		0.985
Deformity	93 (17)	
Oncologic	98 (8)	
Trauma	99 (27)	
**By approach**		0.801
Open	94 (42)	
Minimally invasive	110 (11)	
**By mechanical complication**		0.779
No complication	94 (48)	
Complication	102 (5)	

Each value is the median, across patients in that stratum, of the patient-level mean attenuation measured at L1 through L4. *p* by Kruskal-Wallis (indication) or Mann-Whitney U (approach, complication). The complication stratum contains 5 patients. HU, Hounsfield units.

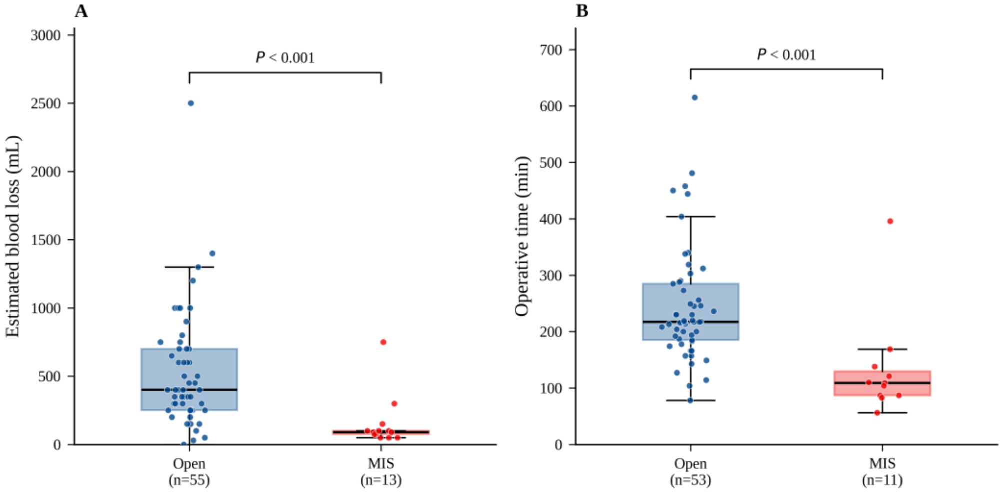

Supplementary Figure S1. Operative metrics by surgical approach. (**A**) Estimated blood loss, open n = 55 and minimally invasive n = 13. (**B**) Operative time, open n = 53 and minimally invasive n = 11. Boxes denote the interquartile range, central lines the median, and whiskers the range excluding outliers. Points are individual patients. *p* values are from the Mann-Whitney U test and are descriptive.
